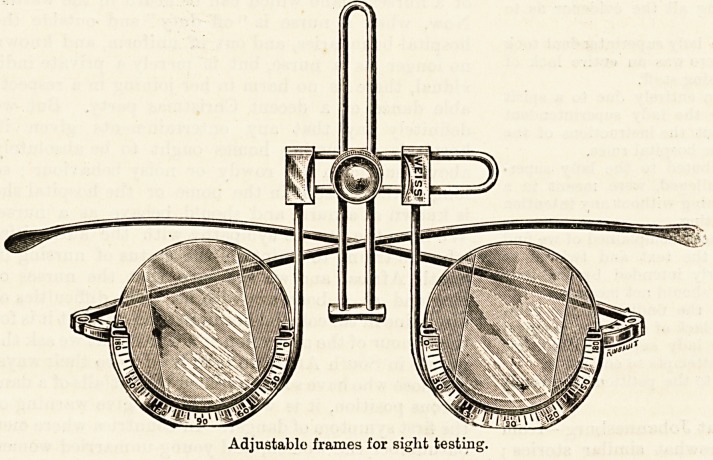# Nursing Section

**Published:** 1903-12-05

**Authors:** 


### The Hospital.

### Nursing Section.

Contributions for this Section of "The Hospital" should be addressed to the Editor, "The Hospital" Nursing Section, 28 & 29 Southampton Street, Strand, London, W.C

No. 897.—Vol. XXXV. *SATURDAY, DECEMBER* 5, 1903.

### Notes on News from the Nursing World.

### OUR CHRISTMAS DISTRIBUTION.

We remind our readers that the latest day for sending in parcels is Monday December 13th. The articles received by us for distribution up to that date will be on view at the offices of The Hospital, 28 & 29 Southampton Street, Strand, on Tuesday, December 14th. Nurses who wish to see for themselves the contributions which have been forwarded to us from all parts of the country for the benefit of patients in hospitals and infirmaries, will be welcome between 3 and 5 p.m. We have to acknowledge, with thanks, further parcels from:—Nurse Rose Sneath, 17 St. Catherine's Road, Grantham; "Anon," Camden Town; Nurse Young, Faringdon, Berkshire; "Owl," district nurse, Streatham; Miss Hall, the Parish Infirmary Portsmouth, and Miss L. Taylor, College Road, Dulwich.

### THE JUNIUS. S. MORGAN BENEVOLENT FUND.

The hon. Secretary of the Junius S. Morgan Benevolent Fund asks us to remind Pension Fund nurses, through our columns, that a very short time remains before the end of the year, and that only 563 nurses have sent a subscription of 1s. to their benevolent fund in 1903. The hon. Secretary knows that so small a matter is easily overlooked by busy nurses. She feels sure, however, that this reminder will meet with a hearty response. The calls upon the Benevolent Fund grow larger every year, and the committee are somewhat apprehensive that they may not be able to meet all the really urgent demands made upon them in the near future, as their resources have been heavily taxed this year, and the list of practically permanent pensioners is so large. Subscriptions are therefore very welcome, and should be sent to the Secretary of the Royal National Pension Fund for Nurses, 28 Finsbury Pavement.

### QUEEN ALEXANDRA'S MILITARY NURSING SERVICE.

Miss E. A. Wilkinson has been appointed matron of Queen Alexandra's Imperial Military Nursing Service, and Miss M. L. Harris staff nurse provisionally. The appointments of Miss M. M. Blakely and Miss M. L. Potter as staff nurses are confirmed.

### RESIGNATIONS AND DISMISSALS AT CHARING CROSS HOSPITAL.

As statements have been made in an evening paper respecting the matron and the nursing staff at Charing Cross Hospital, we think it desirable to give the facts as ascertained by us. On Saturday morning, November 21st, the matron, acting under the order of the nursing committee, asked three sisters and a staff nurse, who was on trial for the post of sister, to come to her office, and, we are informed, invited them to send in their resignations, or she would be compelled to take stronger measures. After dinner on the same day she gave notice to three staff nurses and to a probationer who was performing the duties of staff nurse. All the dismissed sisters and nurses have been on the staff for periods varying from three to seven years. Following these resignations four more of the sisters resigned, entirely from a feeling of sympathy with their colleagues, and not because, as has been alleged, certain alterations have been made in the regulations. It is, we understand, perfectly true that theatre leave has been cut down, the staff nurses being now given leave once a month instead of once a week, and the probationers once every month, but this alteration does not affect the sisters, of whom, however, only two propose to remain at the hospital.

### APPOINTMENTS TO CAMBERWELL INFIRMARY.

The following important appointments have been made to Camberwell Infirmary:—Miss Rosa Wallace has been appointed assistant matron. She was previously superintendent nurse at the Gordon Road Workhouse of the parish. Miss Annie Willis, sister at the infirmary, has been appointed superintendent nurse at the Gordon Road Workhouse. Miss M. I. Cleghorn has been appointed sister at the infirmary; she was previously a member of the Army Nursing Service Reserve, and was trained at the Royal Infirmary, Glasgow, where she was also a charge nurse. Staff nurses Fanny Quilter, Emma Corney, and Emily A. Wright have been promoted to be sisters, and staff nurse Ellen P. Turner has been appointed sister. They were all trained at the infirmary. Miss Mary C. A. Bayliss, trained at Portsmouth Infirmary; Miss Rhoda A. Backett, trained at Brompton and Guy's Hospitals; Miss K. M. Goldsmith, trained at Stapleton Infirmary; and Miss Louisa Taylor, trained at Lewisham Infirmary, have been appointed staff nurses. These appointments show that hospital trained nurses do not, as some of our correspondents seem to think, always carry off the Poor-law prizes.

### THE BISHOP OF STEPNEY ON NURSING.

The opening last week of a home for the nurses in Raine Street, which is described in another column, marks a step forward on the part of the guardians of the important parish of St. George's-in-the East. The ceremony itself was invested with additional interest by a speech from the Bishop of Stepney, who, in performing the function, intimated his conviction that the Home was badly needed. He also expressed a hope that the nurses would realise the dignity of the work to which they were called. He always thought that service was most enjoyed where there was most sacrifice, and there was no doubt that in the particular work nurses had to do there were constant calls for self sacrifice—the sacrifice of petulance, of the instinct of impatience, and of their own comfort. Let them not think that it was nursing that was easiest, which obtained the quickest advance, the highest pay, or the greatest possible notice, which was really the most honourable. The most honourable was that in which the sacrifice was the greatest.

### THE PERILS OF STARTING A NURSING HOME.

No sensible person who reads the authentic account of starting a nursing home, which we give in another page, will be induced by the personal experience of our contributor to face a similar ordeal with the probability of a disastrous conclusion. Of course, we are aware that there are people who, because our contributor, with many circumstances in her favour, was at last successful, may tempt Providence in a similar manner. The majority, however, who possess the gift of imagination and the virtue of common prudence, will see in the forestalment of an income at ruinous interest; in the absence of patients week after week and month after month, notwithstanding all the advantages of a good position and the backing of influential medical men; in the petty shifts to which the unfortunate woman who had ventured upon a perilous enterprise was compelled to resort; in the mortification of dismissing servants because there was neither work for them to do nor money to pay their wages; and in the "daily gnawing despair," which must have been aggravated by the knowledge of a lamentable want of judgment, abundant reasons for refraining from following her example. She herself points the moral by repeating the warning which we have so often given, not to start a nursing home without an ample amount of capital. But it should not only be ample; it should be capital which the owner can afford to lose. No one would think of investing money which she needs, or may need for the necessities of existence, in such a hazardous enterprise as a nursing home.

### A CHANGE OF TITLE.

At the annual meeting of the Stockport Sick Poor Nursing Association, it was decided to alter the title to the Stockport Sick Poor and Private Nursing Association, and we agree that in the circumstances the change was desirable. When the association was formed 12 years ago, it was only intended to minister gratuitously to the needs of the sick poor. But since then private nursing has been undertaken by the organisation, and it is only fair that the public should not be misled by an incorrect title. It cannot, however, be said that the association has any particular cause to congratulate itself upon its departure from its original purpose, for the reports of the two departments show a deficiency on the district branch of £64, and on the private branch of £82. We suspect that a good many persons in Stockport are under the impression that the fees earned by the nurses who attend private patients help to maintain the district branch. The managers of the Stockport Association are, we hope, fully aware of their legal responsibility with regard to the actions of their nurses, and do not share the delusion which until lately prevailed upon the subject at Oldham.

### THE CENTRAL MIDWIVES BOARD AND ITS CERTIFICATE.

At a meeting of the Central Midwives Board on November 26th, letters were read from the Clerk of the Worcestershire County Council and the Medical Officer of Health for the County Borough of Croydon, inquiring whether illiterate women, unable to sign their own names, were eligible for enrolment as midwives under the Midwives Act. The secretary was instructed to reply that the express terms of Section 2 of the Act give the Board no option to refuse an application from an illiterate woman otherwise qualified under the Section. In the case of a claim for enrolment on the ground of having been in *bonâ fide* practice for the statutory period within the Commonwealth of Australia, the Board decided that as there was nothing in the Act to limit the area of practice to the United Kingdom, the claim might be admitted if otherwise eligible. An application of the Dundee Maternity Hospital for recognition of its certificate as an approved qualification under Section 2 of the Act, claims for enrolment on the qualification of holding a certificate from Sir Patrick Dun's Maternity Hospital, Dublin, the Hospital for Women and Children, Lupus Street, S.W., and applications for recognition as approved institutions for the training of midwives from the District Nursing Association, St. James's Square, Cheltenham, the Maternity Charity and District Nurses' Home, Plaistow, and the Brighton and Hove Hospital for Women, together with others already received, were referred to the Standing Committee for consideration and report. After consideration of applications for certificates, the names of 213 women were passed under Section 2 of the Act, and ordered for entry on the roll. Of this total 61 claimed as holding the certificate of the Obstetrical Society of London, four that of the Rotunda Hospital, Dublin, five that of the Glasgow Maternity Hospital, and 143 were admitted as having been in *bonâ fide* practice for one year prior to July 31st, 1902. Draft rules of procedure on the proposed restoration to the roll of a name removed were considered, amended, passed, and ordered to be submitted to the Privy Council for approval.

### MONUMENT TO SPANISH-AMERICAN WAR NURSES.

At the fourth annual meeting of the Spanish-American War Nurses in Tresidio, San Francisco, a statement, embodied in the report, respecting the progress of the movement for the erection in the National Cemetery at Arlington of a memorial in memory of the nurses who died in the war of 1898, was read, and it was subsequently resolved that the society should proceed as soon as possible to carry out the proposition. It was decided that the monument fund should be increased to at least 3,000 dollars, and that a committee should be elected to select a sculptor. As it is more than five years since the five nurses sacrificed their lives in the discharge of their duties, the recognition of their devotion is rather tardy. Various reasons for the delay are suggested, but memorials should be proceeded with while the events intended to be commemorated are fresh in the minds of the people.

### ALLEGED "MENACING LETTERS."

THE principal business discussed at the last meeting of the Grimsby Board of Guardians had reference to the work done by the various nursing associations of the town. The matter had been debated at some length in Committee with the result that it was decided to recommend the Board to make the following grants: Bargate Institution, £40; St. Xavier's, £15; Wesleyan Association, £10; Hospital, £20; Waltham Institution, £5. In the course of the discussion the Chairman said that they had received a menacing letter which practically said "Unless you give us so much we shall not do anything for you." This was not the only case in the country. There were ten or a dozen Unions which had been subscribing to institutions of this kind but owing to menacing letters from these institutions these Unions had withdrawn the subscriptions entirely. "It doesn't matter," said the Chairman, "when you receive menacing letters of that description. These are private institutions, and are not under the control of the Board in any way whatever." The statement of the Chairman was challenged by several members, who asked him to produce the names of two of the nursing institutions which had sent "menacing" letters. The Chairman said he had not got the names but he could give those of a dozen. The result of the debate was that the Bargate Institution will receive £50, the St. Xavier's Institution £15 15s., the Old Dock Road Mission £10, and the Hospital £25.

### A PIANO FOR WORKHOUSE NURSES.

THE Christchurch Guardians have had under discussion a letter from the superintendent nurse requesting that a piano might be provided for use at the nurses' home attached to the workhouse infirmary. The clerk being asked if he thought that the Local Government Board would object, replied in the negative; and it having been urged that the nurses had very little recreation after duty, it was decided to purchase a piano at a cost of £20. We conclude, from this limit, that the guardians have in view the acquisition of a second-hand instrument.

### RETROGRESSION AT LOUGHTON.

THE people of Loughton cannot be congratulated upon the support they give to the Queen's nurse who is at work among them. Last year, out of a population of nearly five thousand, only 129 persons subscribed to the District Nursing Association; the total amount contributed fell from £79 12s. 6d. to £72 4s.; and the sum collected in the churches and chapels was only £12 14s. 6d. as against £21. Yet the number of visits paid by the nurse was 2,831 compared with 2,763 last year, and Miss Peter, the general superintendent, reported that she had performed her duties in a very satisfactory manner. There the cause for satisfaction ends.

### QUEEN'S NURSES AT LOUGHBOROUGH.

AN interesting incident at the seventh annual meeting of the Loughborough Queen's Nursing Association was that the Rev. C. B. Bartlett, of Thorpe Acre, referred to the work of the nurses during an outbreak of disease in that village. He said that he could testify to the very great help and comfort they had been. It is pleasant to learn that the villagers who derived benefit from the nursing manifested their gratitude by contributing to a small fund raised by the churchwardens and sent to the association. The entire number of visits paid by the nurses during the year was 5,264, the number of cases visited being 171. The inspector for the district reported the work "very satisfactory." In one respect the financial position is secured, the balance of income over expenditure being nearly £26. But, on the other hand, the regular smaller subscriptions have slightly decreased, and as extra expenditure will soon become inevitable, it is obviously requisite that these should be increased.

### NURSING HOMES AND THE COMPANIES ACT.

AMONG the limited liability companies registered in November is the Yorkshire Co-operation for Nurses and Nursing Homes. The object is to acquire the premises of nursing homes for patients undergoing massage and medical, surgical or other treatment, and for maternity cases, carried on at 22 and 24 Clarendon Road, and 9 and 15 Hyde Terrace, Leeds, by Alice M. Wall, Kate Atkinson, and Edith A. Woodcock. The capital is £5,000 in £10 shares, and there is no initial public issue. The names of the first directors, to number not less than seven nor more than nine, are not given; but the qualification is £50.

### WHAT'S IN A NAME?

SOME opposition was offered by members of the St Olave's Board of Guardians to the appointment of a lady as assistant matron of the Parish Street Workhouse on account of her name—Blanche Zytogorsky; but as her qualifications were the best she secured the office.

### SHORT ITEMS.

A CONFERENCE under the auspices of the Association for Promoting the Training and Supply of Midwives was held at 2 Cromwell Houses, S.W., on Wednesday afternoon, Lord Monkswell in the chair. The proceedings will be reported in our columns next week.—The Town Council of Luton are looking after the welfare of their nursing staff at the Spittlesea Isolation Hospital. Through the recommendations of the sanitary committee they have caused to be erected a covered way between the administration block and the scarlet fever wards, which the nurses very much appreciate.—An entertainment, organised by the "Pinero Dramatic Club," was given to the in-patients of the Cancer Hospital, Fulham Road, London, on Thursday last. The farcical comedy by Sidney Grundy, entitled "The Snowball," was acted, and a hearty vote of thanks was accorded on the proposition of the secretary at the termination of the piece.—At the recent examination of the Obstetrical Society of London, for the midwifery certificate, the following nurses, who received both their general and midwifery training at the Walton Workhouse and Infirmary, Liverpool, were successful: P. Eastham, F. Hill, A. Parlby and F. Spencer.

### The Nursing Outlook.

"From magnanimity, all fear above;

From nobler recompense, above applause,

Which owes to man's short outlook all its charm."

### THE NURSE IN SOUTH AFRICA.

Now that the war is over it seems there is a certain reaction and demoralisation in many ways in South Africa—and nurses have not escaped the contamination. Four months ago we gave an account of the state the nursing was in at Johannesburg when the Committee called for an English matron to go out: last week we gave an account of what is very like a rebellion on the part of the nurses. Luckily the Committee are strongly on the side of reform, and after a full investigation by a special committee of the nurses' complaints, they have issued the following report:—

The Hospital Board, having considered the report of the sub-committee appointed to investigate the complaints made by the nursing staff, unanimously agreed that the same be adopted, and that the nurses in question be furnished with a copy as follows:—

The committee find, after hearing all the evidence as to facts, as follows:—

1. That at the time when the lady superintendent took charge of the hospital there was an entire lack of discipline among the nursing staff.
2. That all the complaints are entirely due to a spirit of resentment caused by the lady superintendent endeavouring to carry out the instructions of the Board, as laid down in the hospital rules.
3. That the utterances attributed to the lady superintendent, if made as alleged, were meant in a spirit of advice and warning without any intention of being personal or insulting.
4. That many of the utterances complained of we find have been taken from the text and twisted to mean what it was clearly intended by the lady superintendent that they should not mean.

The Board are of opinion that the finding of the committee shows that there is a great lack of discipline amongst the nursing staff, and that the lady superintendent has been consistently opposed in her attempts to enforce it.

We resolve that the signatories to the petition be severely reprimanded for their action.

But the trouble is not only at Johannesburg—from all the big towns we get somewhat similar stories; everywhere the nurses seem to be given over to frivolity, and the nursing staffs to lack discipline and dignity. For instance, the nurses of the New Somerset Hospital, Capetown, having taken part in a fancy dress ball, where some of the nurses appeared in male costume, and one of the doctors in female costume, complaints were sent to the committee not only as regarding the ball, but also as regarding other entertainments given by the nurses. The result was a long and severe investigation at the end of which the matron and the assistant matron, and one of the medical officers were asked to resign, and offered their passages to England. The matron then sent in the resignations of six sisters and 13 probationers, and called in the services of a lawyer. The committee have stood firm and have appointed another matron. Of the tone of the malcontents we need no further evidence than that published in the *Owl* of September 25th, in an interview with the dismissed assistant matron:

The assistant matron replied that it was difficult to definitely enumerate them; the allegations were of such a vague and miscellaneous description. It was said that at the last Christmas dinner their behaviour was rowdy and noisy, and that at the dance afterwards they did not conduct themselves with due decorum. One statement was that the matron dragged a nurse round the room by the leg. As a matter of fact, the nurse who was alleged to have been so treated did not attend the dance. Again it was said that they imbibed champagne too freely. Now the "fizz," it was interesting to note, was the gift of one of the house surgeons, and consisted in all of fifteen small bottles. One third of the quantity was presented to Dr. Moffatt, so that ten small bottles among a party of about a score could hardly be said to be an excessive supply. A further insinuation was made that some of their costumes at various dances held during the year had not been "nice" forsooth! There was another little function which formed the subject of complaint, namely, the fancy dress ping-pong tournament, held no less than 16 months ago. As to the fancy dress ball which had led up to the so-called "scandals," the assistant matron pointed out that the sister who helped to make mischief was present and she said she had enjoyed it very much!

Here is a glimpse of what South African nurses consider quite fit and proper behaviour in a hospital devoted to the care of the sick and dying. A third report that lies before us is a protest, because the medical officers have tried to stop the continued use of a nurses' piano which can be heard in the wards! Now, when a nurse is "off duty" and outside the hospital boundaries, and out of uniform, and known no longer as a nurse, but is merely a private individual, there is no harm in her joining in a respectable dance, or a decent Christmas party. But we definitely say that any entertainments given in hospitals or nursing homes ought to be absolutely above suspicion of rowdy or noisy behaviour; so long as the nurse is in the home or the hospital she is known as a nurse and should behave as a nurse. We have the utmost sympathy with the authorities who are trying to maintain the status of nursing in South Africa, and we are sure that the nurses of England as a body are with us. The difficulties of discipline in the colonial hospitals are great, but it is for the honour of the profession as a whole that we ask the nurses in South Africa to take heed as to their ways. To those who have so far avoided the pitfalls of a dangerous position, it is worth while to give warning of the first symptom of danger. In countries where men outnumber the women, and young unmarried women are specially sought out and flattered, vanity is the first temptation. But jangling bracelets and sparkling rings are out of place with a nursing uniform, and no dress is beautiful which is not appropriate. The charm of the nursing uniform lies solely in its simplicity and suitability. Then vanity leads to the encouraging of addresses which are in reality far from flattering; it leads to egoism and selfishness and forgetfulness of patients; it leads to pride, insolence, and ignoring of authority; it leads to rudeness, rebellion and dismissal. Further we do not care to follow, for the fate of the vain, self-centre woman who is thrown out of work in a colonial country is generally swift and hopeless. At the best she becomes the wife and general servant of some settler in the wilds, and drudges on day after day, cut off from all intellectual and elevating companionship, cut off from all the comforts and amusements of civilisation, and doomed to a worn out and early old age. In conclusion, how is it that registration has not cured all these nursing scandals in Cape Colony?

### Lectures on Ophthalmic Nursing.

By A. S. COBBLEDICK, M.D., B.S.Lond., Senior Clinical Assistant and late House-Surgeon and Registrar to the Royal Eye Hospital.

### LECTURE XXIV.—DISEASES OF THE VITREOUS, RETINA, AND OPTIC NERVE.

DISEASES of the vitreous and fundus are a frequent source of impaired vision, an important feature of the impairment being the inability to improve it by any glass or combination of glasses

*Diseases of the Vitreous.*—The most common trouble is hæmorrhage. This takes place suddenly from rupture of a small retinal vessel; patients frequently state that they notice it for the first time on awaking in the morning, and describe the condition of vision as like trying to see through a fog.

The impairment of vision varies directly with the size of the hæmorrhage: in severe cases fingers cannot be counted, but moving shadows may be detected, even in comparatively slight cases $\frac{6}{60}$ cannot be placed to the patient's credit.

There are no other marked symptoms.

On inspection of the eye but little can be seen without the

Adjustable frames for sight testing.

use of the ophthalmoscope; by using reflected light and comparing the diseased eye with the healthy one a marked difference is noticeable: a red reflex (the fundus reflex), due to the vascularity of the retina and its coverings, is at once manifest in the sound eye, whilst it is absent or very faintly marked in the diseased one. On looking at the affected eye more closely no details of the fundus such as the disc-vessels or macula can be seen.

*Prognosis.*—Slight cases do very well, as the extravasated blood becomes absorbed in the same way as in other parts of the body. Severe cases clear up to a certain extent, but leave some opacities and cause considerable defect of vision.

Severe cases, and where recurrence of the hæmorrhage takes place, are mostly associated with retinal and choroidal changes, so that the latter may be the chief cause of loss of vision.

*Treatment.*—The first essential is to lower the blood tension and so diminish the risk of further hæmorrhage, and the second is to promote absorption of the extravasated blood, and so improve the vision. When the first cause of the disease can be traced to constitutional disease, this must be treated. The blood tension is lowered by purgation, rest, and low diet. The best purge is a saline in the morning, or a dose of calomel, gr. ii. to v. at night, followed by a seidlitz powder or one of the mineral waters, such as Hunyadi Janos or Apenta water. Absorption of the hæmorrhage is best promoted by the use of potassium iodide, and at the same time, if syphilis is the original cause, mercury may be added to the mixture in the form of the liquor hydrarg. perchlor. ʒi. doses.

The other most important affection of the vitreous is the presence of a foreign body. This may or may not cause trouble; as a rule it sets up severe inflammation, involving the whole contents of the eye and ending in disintegration (panophthalmitis); occasionally the inflammatory trouble settles down and the foreign body causes but little harm. Pieces of steel and iron are the commonest forms of foreign body and they are the most easily treated, as they can usually be extracted with the electro-magnet.

The cysticercus (hydatid), if deposited in the vitreous, acts as a foreign body and sets up a severe inflammation

The only diseases of the retina which will be noticed in these lectures are:—Inflammation of the retina (retinitis), detachment of the retina, and glioma of the retina.

Retinitis is almost always a sign of some constitutional disease, of which syphilis, albuminuri diabetes, and leucocythemia ar the most common.

The symptoms consist of considerable loss of vision and inability to face a strong light (photophobia). As a rule both eyes are affected.

The inflammation causes the retina to swell, to become hyperæmic, and to set free an exudate which passes into the vitreous and causes *opacities*; thus the signs are much like those of an inflammation elsewhere.

The swelling of the retina is most marked near the optic disc and may be so great as to obscure the blood-vessels for a short distance.

Another result of the hyperæmia is hæmorrhage, in the form of elongated patches, for the most part contiguous to the vessels.

*Detachment of Retina.*—This is a very serious affection, at times ending in total blindness.

The chief causes are:—(i.) High degrees of short sight (high myopia), in which condition the vitreous undergoes a degeneration.

(ii.) Exudations into the vitreous caused by irido-choroiditis or irido-cyclitis.

(iii.) After considerable escape of the vitreous following an injury or operation; or tumours of, and hæmorrhages from, the choroid.

All these causes produce a change of balance in the pressure on the two sides of the retina, and as the latter is not firmly attached to the choroid, but only, for the most part, laid on it, it (the retina) is easily displaced.

*The symptoms* consist of sudden loss of vision in some part of the visual field; the tendency is for the detachment to increase, but as long as the macula is intact, central vision, may remain good. In total detachment there is complete blindness.

With the ophthalmoscope the detached portion is greyish white in colour; it is pushed forward into the vitreous and the blood-vessels are small and attenuated.

*Treatment.*—This consists of complete rest in bed, purgation, and the administration of the iodides. Removal of the subretinal fluid by puncture of the sclera has been performed with some success, but, unfortunately, recurrence of the detachment takes place. What is required is something to rejuvenate the degenerated vitreous.

When the detachment can be ascribed to a new growth, the eyeball must be enucleated without loss of time.

*Glioma of the Retina.*—This is the only form of tumour which occurs in the retina. It always appears in childhood, usually before the fifth or sixth year, and is a most serious affection, causing certain loss of the eye and possible extension of the growth backwards by the optic nerve to the brain, and to other vital organs by means of the lymphatics.

The growth is limited to one eye; it causes complete blindness in the affected eye, but in the early stage there is no pain. As the growth forms and presses forward the tension of the eyeball increases, and it becomes painful and irritable. The treatment consists in the prompt enucleation of the eye, with division of the optic nerve as far from the eyeball as possible.

### History of a Nursing Home.

### A PERSONAL EXPERIENCE.

My personal experiences as proprietress and matron of a nursing home in London, will, I think, assume a particular interest in the eyes of many of your readers when I preface my story by saying that at the time I started my home I was in receipt of a small annual income from some landed property in Ireland. To provide means of living for a short time, I anticipated this income through a money lender, at ruinous interest. For obvious reasons I can give neither the name nor the locality of my home, yet I may mention that it was situated in a fashionable, healthy, and bracing district of London. A good healthy position, is, by the way, essential, when deciding upon the site for a home.

### Promises of Good Work.

Before deciding on my house I obtained from mutual friends introductions to most of the chief physicians and surgeons in my desired neighbourhood. I had of course introductions to many doctors, specialists, etc., who were outside this radius, but, as a rule, I found that doctors, if they can avoid it, do not like to send their patients to homes not in the immediate neighbourhood of their own residences. Nearly all my "introduction" doctors said that when they had work for a home they would be glad to give it to me. This, however, was only a conditional promise, and on its sole responsibility I would not have started a home had I not received from two or three surgeons absolute promises of good work. This luck was in consequence of a lady having lately closed up her well-liked home in the neighbourhood, leaving a void which I was given a chance to fill.

### A Costly Mistake.

My next step was to secure a house. I decided on one large and solidly built, of which the rooms were all light, airy, and of a good size. Moreover it was well situated right in the midst of the doctors. But I made one fatal mistake, which cost me sixty hard-won pounds to rectify. I entered into possession of the house without having obtained an "independent" sanitary certificate. By "independent" I mean a certificate given after careful examination of the premises, by a certified sanitary officer, not employed by the London County Council nor governed by their rules. I mean no discourtesy to the London County Council when I make this statement. I make it only in consequence of the fact that many doctors, as they did in my own case, insist upon nursing homes obtaining, plus the London County Council certificate, the more particular and minute certificate of an independent sanitary officer. This independent certificate costs two guineas, and it should be obtained before an agreement is signed. In that case you can, of course refuse to take the house if the independent man insists upon alterations whose necessities were passed over by the London County Council. After you have taken possession the landlord is only bound to satisfy the London County Council, independent alterations must be paid for out of your own pocket.

### The Patients' Rooms.

All the walls of the rooms to be used for patients were distempered, not papered. The colours chosen for distemper were soft, restful greens—which decidedly take first place on account of their good effect on patients' eyes—pale blue, and pale pink. The floors of these rooms were covered with cork linoleum—cork linoleum being chosen both for its freedom from germs of infection, and for the ease and celerity with which it may be washed and dried. The patients' rooms contained, and should always contain, very little furniture. Mine, by a doctor's advice, was all plain white enamelled. I generally found that a white enamelled combination washhand-stand and chest of drawers, a few plain chairs and an easy one, a couple of rugs, a small table for the patient's bedside, a large three-portioned screen of the same shade as the walls, and of course a night chair, a thermometer, and the ordinary toilet utensils, were, including the bed, the only articles absolutely required for a patient's room. Clothes, trunks, or boxes of any kind were never allowed to remain in the sick-rooms, but were kept outside in wardrobes, etc., specially constructed for them, the keys of which were kept by the matron. All my furniture had unfortunately to be obtained on the hire system, and I had good cause to realise how disheartening it is to commence an enterprise with a load of debt.

### Purchasing Appliances.

I did not purchase many surgical appliances or drugs at first, but on different cases coming in, bought them as I required them. Everything of this sort, including all nursing vessels, dressings, patients' medicines, instrument trays, mackintoshes, and mineral waters, etc., were obtained from the same chemist, with whom I had arranged for a good discount to be allowed me on all my orders. As I could not possibly afford to fit up a theatre, I had an operation table made, which was carried to and from the patients' rooms for operations, and at other times kept covered over in a partitioned part of the bath-room, where hot-water bottles, mackintoshes, disinfectants, measuring-glasses, etc., were also stored. A few gas rings were here, too, fitted up for the nurses' use and for boiling instruments. But all medicines, drugs, dressings and bandages of all kinds were kept in locked cupboards on the chief corridor. As for the operation table, it was of plain deal, seven feet by three, and by my order had "rests" for its legs, so that it could be either raised or lowered as the operating surgeon desired. I also made large mackintosh aprons, something of the shape of butlers' aprons, for the doctors' use during operations. These aprons, protecting as they did, the surgeons' clothes from all risks of stains or splashes during their work, I found always much liked and appreciated.

No Patient for Four Months.

I had now got my home all in readiness, even down to the flowers in the drawing-room which I reserved for interviewing patients. I had another drawing-room for the use of my convalescent patients and their friends. A third room was furnished as the family and nurses' dining-room. And now commenced my long, dreary acquisition of that mournful knowledge "that hope deferred maketh the heart sick." For during four long months not a patient's shadow ever darkened my doors. I cannot even yet bear to think of that awful time. I will only say that I would not wish my worst enemy to be the penniless proprietress of a struggling nursing home, a home started without capital. I had engaged the necessary servants for my home—one after the other I was forced to dismiss them, till not one was left. My daughter and I did the cooking (when we had anything to cook), and also the necessary house work. Ah me! the daily gnawing despair, the hopeless struggles; finally, the worn-out numb indifference to failure. Will the pitiless pain of their remembrance ever allow of gladness to spring in my life again? Finally, one day when I had arrived at that point where "the last straw breaks the camel's back," and I had just exhausted the money I had raised, which had kept us from actual starvation, I was saved—my first patient came to the home!

The First Patient.

She was a Weir-Mitchell case, and one of the worst that I have ever known—in fact, she was on the verge of certified insanity when she came to me. Day and night I nursed her myself; she nearly cost me my life, but she made a perfect recovery! And with her my luck changed; other patients followed (my second case was a man—appendicitis), and at last I found myself standing in that envied, but dangerous situation, the ante-room of success! I engaged a permanent staff nurse, to whom I paid a yearly salary, and under her command I had one, two, three, or four nurses, just as the number of my patients required them. This plan, though personally I preferred it to keeping three or four nurses even when their services were not required, I by no means recommend to Hospital readers, for it is far from economical. I advise them instead to engage, according to the size and working order of their home, all their nurses permanently; though of course, I except the "emergency" nurse, whom even the best regulated homes will occasionally require.

The Terms and Meals.

My terms for patients averaged from four to twenty guineas per week, but rarely as low as the former, not often as high as the latter. Night or special nurses were of course always among the "extras" of the home. The patients' hours of meals were as follows: 8 A.M., breakfast; 8.30 A.M., toilet, medical care, dressings, etc.; 10.30 A.M., beef-tea, milk, etc. (according to doctor's orders); 1 P.M., dinner; 4 P.M., tea; 7 P.M., supper; 7.30 P.M., toilet, medical care, and dressings for the night; 9 P.M. milk, beef-tea, etc. (according to doctor's orders); 9.20 P.M., lights lowered for night. Food given during the night was generally prepared by the night nurses, who came on duty at 9.30 P.M. The doctor's visits were paid at quite irregular hours. The same remark applies to operations, though I am glad to say that most of ours took place between 8 and 10 A.M.—by far the most convenient hour for a home.

Visitors and Accounts.

The visitors' hours were from 2 to 4 P.M. I allowed them at no other time, and I would never have allowed them at all if I had possessed the power of refusing them, for it was only the best of the visitors who refrained from worrying every nurse they came across. They were, of course, always successful in agitating and unnerving their sick friends. Indeed, I think most nurses will agree with me when I say that patients' visitors cause infinitely more trouble than the patients themselves ever do. My daughter, who assisted me greatly, always kept books containing a careful account of the income and expenditure of the home; these books were checked every three months by a chartered accountant, and afterwards I found that their existence greatly facilitated the remunerative sale of my home, for at the height of its success fate called me to the bedside of my wounded son in South Africa, and I disposed of my home in a most satisfactory manner. To those who wish to follow in my footsteps, lest their struggle with adverse circumstances and dreary waiting should have a less happy termination than mine, let me add once more, don't start a nursing home, however good your connection may be, without an ample amount of capital!

### The Nurses of St. George's in the East.

The nurses attached to the infirmary of St. George's-in-the-East, who have been up to the present most inadequately housed, have just moved into the new quarters provided for them by the Guardians at a cost of some £12,000. The new building is immediately opposite the infirmary, with which it is connected by a subway, and is flanked on two sides by a small garden with asphalt paths. The main entrance is by a covered porch, and the hall is both spacious and well lighted. The entrance floor is devoted to the matron's office, sitting-room and dining-room, sisters' and probationers' sitting-rooms, staff messroom, and kitchen, etc., while one end of the long passage is devoted to lavatories and cloak-rooms. The colour chosen for passages and staircases is a soft pale green; that in the matron's rooms is cream; while the sitting-rooms of sisters and probationers are of a pale shade of terra-cotta, selected with a view to imparting warmth and cheerfulness even on dull days. Comfortable chairs and sofas, deep window-seats, a green-tiled fireplace, and a good piano make the probationers' sitting-room a particularly pleasant and inviting apartment, while the door leading directly into the garden will be a special delight during the summer. The view of old houses in process of demolition is the least attractive feature, but the planting of a row of trees gives promise of a degree of privacy unusual in the heart of the slums. The London County Council recently closed a row of small houses, in order to give an extra width of 6 feet to the nurses' garden. Lockers for the night nurses are provided in the mess room; and on the basement is a bicycle-house and ample room for boxes, which are easily accessible to the staff. A sloping path precludes the necessity of carrying bicycles down stairs.

The three upper floors are occupied by sitting-rooms for the assistant matron, home sister, and night superintendent, and by the bedrooms—44 in all. These are small but comfortable; the floors are covered with thick cork floor-cloth, the beds have wire-woven mattresses, and the furniture consists of washstand and dressing table combined, a hanging press, and chairs. The sisters' rooms are, in addition, provided with easy-chairs. Picture rails have been fixed in order to avoid nails in the walls. Each nurse has a separate room. One end of each corridor is occupied by bath-rooms and lavatories, and a gas-stove provides for the inevitable emergency cup of tea. The colouring is terra-cotta and pale heliotrope on alternate floors, and the windows on one side of the Home are fitted with ground glass, these rooms being somewhat overlooked. The building is heated throughout by hot-water coils, the floors are fireproof, and there are two main staircases.

### Anglo-American Nursing Home, Rome.

STATEMENT BY THE MANAGING COMMITTEE.

We have received the following letter:—During the summer months of this year, letters and communications have appeared in your journal criticising the administration of the Anglo-American Nursing Home in Rome. They have dealt with two matters; in the first place with an alleged letter of complaint against the management drawn up by the nurses employed, and secondly with a specific complaint of an individual nurse in regard to her own particular case.

So far as concerns the general charge of complaints made by the nurses, this is somewhat ancient history, and goes back to the season 1901–1902. The document in question, which the Committee had good reason for believing not to be a spontaneous production of the nursing staff, contained, not so much complaints, as a number of suggestions for internal improvements in domestic arrangements. Some of these, which had already occupied the attention of the Committee as desirable in themselves where funds permitted, have been adopted, others were rejected. In every case the matter formed the subject of investigation, and the action of the Committee in this respect has received the approval of an overwhelming majority of the subscribers at a general meeting.

As regards the allegation that the Anglo-American Nursing Home will henceforth encounter difficulties in securing a competent staff of nurses, it will probably suffice to mention that of the 11 nurses engaged last season, seven applied for re-engagement during the present season, and that for the vacant places, there were over a hundred candidates from whom a very careful selection has been made.

With regard to their general management, the Committee do not feel called upon to defend the action which has received the cordial approval and support of the subscribers. A small dissentient minority have, however, now addressed their criticisms to your valued journal and have even, it appears, taken pains to circulate these criticisms in the form of cuttings among the matrons of hospitals in England. Surprise has also been expressed that no notice has been taken of these communications. It must, however, be perfectly well-known to your correspondents that the Anglo-American Nursing Home, which has not yet sufficient funds to remain open all the year round, is closed during the summer months, and that the Committee are, at that season, for the most part, absent from Rome. The moment chosen for initiating this campaign would therefore seem to have been purposely selected. It was not possible to assemble a meeting of the Committee at which cognisance could be taken of these communications until November.

The letter which appeared in your number of September 5th, from "An English Nurse," then came under their notice. In this letter, the nurse, who will be referred to as X., states that while nursing a patient, who will be referred to as A., she was laid up with a severe attack of typhoid in a "thoroughly Italian mountain place without reliable doctor or chemist," and, after being treated with reprehensible neglect by the authorities of the home, was finally charged with breaking her contract.

It will, perhaps, be a matter of some surprise to those who have read this letter to learn that the "mountain-place" in question, was the neighbouring town of Tivoli, having a population of over 10,000 inhabitants, and the object of daily excursions from Rome by tramway, train, and carriage. A short history of the case will no doubt suffice to give the facts their proper relative importance.

Nurse X., who was especially chosen by Miss A., the patient, with whom it appears she had been previously intimate, took charge of her case from November 23rd, 1902, until the end of February last in the Nursing Home. The case was reported to be a light one, except for a brief period in December, when the attendance of a doctor was necessary; so much so indeed that nurse and patient were able to take long drives together when the weather permitted, and even to visit a theatre. On February 25th they went to spend the day at Tivoli, where the patient said she might remain for the night in order to avoid fatigue. The following day Nurse X returned, announcing that Miss A. had decided to remain at Tivoli, and she had therefore come for her luggage. From that time until April 24th indirect news of nurse and patient reached the home from visitors to Tivoli, and from a correspondence, renewed at intervals, between the matron and Miss A. on the subject of her account, which was overdue. On April 24th Nurse X. asked to be released from her engagement, which ran till the end of the nursing season, on the ground that she was wanted at home.

Her letter, referring to a picnic on the previous day, conveyed the impression that her services at Tivoli as a nurse could be dispensed with. The superintendent, who was at the time daily obliged to refuse cases for lack of nurses, replied that she did not feel justified in reducing the staff at that moment. The home did not close until July 14th.

A few days later, Miss A. telegraphed that Nurse X. was ill. The matron at once recommended her to return to the home. Tivoli is only 18 miles distant, connected with Rome, as said before, by train and tramway, as well as a good carriage road. Miss A. replied on April 30th that it was not at all necessary that Nurse X. should return, as she was quite well able to look after her. Then on May 3rd a letter was received from Miss A. announcing that she had on her own responsibility called in a doctor to see Nurse X., as she did not seem better. The doctor, whose identity was not mentioned, had said she was suffering from gastritis and had better remain in bed. Two days later, one of the leading foreign practitioners in Rome, who himself makes use of the home and is frequently there, informed the matron that he had been to Tivoli at the request of Miss A., and that, as she wished it, he should go again. He added that Nurse X. would be in Rome in a week. The matron, who was at the time extremely busy with her duties, was unable to go to Tivoli, but from the report given her by the doctor she was justified in considering the case was not a serious one, "and in fact, on May 10th, Nurse X. was reported to have had solid food—viz., chicken." In the same letter in which this announcement was made, as well as on a postcard written on May 7th, Miss A. stated that the doctor said it was a case of typhoid fever. This was not in accordance with the report given by the medical authority in question, whose certificate, stating that Nurse X. had been suffering from gastro-intestinal catarrh, is in the possession of the Committee.

Had Nurse X. returned to the Home when she first felt unwell, she would have received free medical attendance and every other advantage. Miss A., however, who claims to be herself a trained nurse, declared, on April 30th, this to be unnecessary as she could look after herself, and on May 3rd she summoned from Rome one of the principal resident doctors without any reference to the superintendent, who could have been consulted by telephone, thus taking upon herself the responsibility for the case, which in fact she, in her letters, acknowledged. Miss A. also wrote to the superintendent that she had kept Nurse X.'s family fully informed as to her progress. As Miss A. was in a better position to give them information than the superin-

-tendent, and as the doctor had completely reassured her as to the case, the superintendent did not write herself.

On May 19th the doctor wrote a certificate to the effect that Nurse X. was convalescent, but that she would be too weak to resume nursing duties. On the 20th, Miss A. came to the Home to fetch some effects belonging to Nurse X., but did not see the superintendent or leave any letter or message for her. On the 21st Miss A. and Nurse X. left Tivoli and started for Germany.

Though Nurse X was able to undertake this long journey, she did not either call at the Home, or let it be known that she was passing through Rome, and she left Italy without asking for permission or sending any kind of notification whatever. On the 24th Miss A., who was still largely in debt to the Home, wrote from Germany, asking for Nurse X.'s salary for the last month, and for the money for her journey. On the 28th the superintendent wrote to Nurse X. for explanations, and no answer having been received, a further letter was sent on June 20th. In a letter from Miss A., undated, received June 16th, she announced that she had instructed her bankers to pay a portion of the debt she had contracted towards the Home, the balance of her account amounting to upwards of £30; she was, she said, withholding until she learned what steps the Committee intended to take with regard to the expenses she had incurred through Nurse X.'s illness. Of these expenses no statement was offered. The Committee, after consulting their legal adviser, decided not to pay the expenses of Nurse X.'s journey to Germany. The balance of Miss A.'s account is still owing to the Home.

The Committee of the Anglo-American Nursing Home cannot pretend to be experts in hospital management, but they give a great deal of time and thought to an institution in whose welfare they are deeply interested. In so doing, they have received the cordial support of nearly all the subscribers resident in Rome, who do not consider their efficiency impaired because a member of the British Embassy, the Consul-General of the United States and the British Consul are among their number. (See The Hospital of August 22, 1903.) While considering the efficiency of the house and the welfare of the patients their first care, they feel that they are answerable only to the general body of subscribers to whom they are ready at all times to give every information, and they greatly regret that a few individuals, however well-intentioned, with whose personal views they are not in agreement, should have taken a course which is calculated to impair the utility of an institution whose beneficent work is attested to by legacies, spontaneous donations, and testimonials from medical men and patients, which are open to the inspection of all who may be interested.

The members of the Managing Committee of the Anglo-American Nursing Home in Rome, Italy.

Charles A. Lamb, Chairman of the Managing Committee A.A.N.H.
Lilias G. Rodd.
Maria D. Abbott.
Hector de Castro, U.S. Consul-General.
A. Chenevix Treskle.
Charles C. Morgan.
Richard C. R. Young.
S. Nicholas Vansittart.

Rome, November 25th, 1903.

### Where to Go.

Evening Concert, Queen's Hall, Monday, December 7th, at 8 p.m., in aid of the Seamen's Hospital, Greenwich. Tickets 10s. 6d., 3s., 2s. 6d., and 1s.

### A Training School for Nurses in Naples.

The Jesu Maria Hospital is situated in a very crowded quarter of Naples. In stands in an old garden which seems quite out of keeping with the noise and bustle going on only a few yards away. Very characteristic of modern Naples are the immediate surroundings of the hospital. Up-to-date electric tramcars and gaily-harnessed carozellas, mingled with carts of every description, driven by an equally motley variety of picturesque Italians, are incessantly passing its windows, while the air is filled with the hundred and one street cries peculiar to Naples. Once within the building all is quiet and peaceful, and a great calm succeeds the roar of the outer world.

### The Wards and Operation-Room.

The hospital contains about 230 beds and, apparently, all kinds of surgical cases are admitted, with a lesser variety of medical ailments. The wards have from sixteen to twenty beds each, the floors are of dark-blue stone, and the beds are covered with blue quilts. As is the case in most Italian hospitals no decoration or warmth of colouring is attempted, so that an air of dreariness pervades the wards and leaves a sombre impression upon the visitor's mind. The operation-room is rather small, but a great deal of good work is done in it notwithstanding, and upon the occasion of my visit the solitary English nurse on the staff was busily preparing for forthcoming operations.

### The Training School.

To the Jesu Maria Hospital is attached the only training school for nurses in Naples. Here the pupils undergo a course of training lasting two years. The usual number receiving instruction at one time is twelve; they do not reside in the hospital, but must provide their own board and lodging. No salary is paid them during their training, the teaching provided being considered equivalent to the value of their services. The pupil nurses may leave the wards when their duties are fulfilled, and they are never expected to be in the hospital after 6 p.m. Except in the case of severe operations, they are not called upon to do night duty. All the menial work of the wards is done by Camerieras, a class of women on a level with English wardmaids, who wear a uniform, and render minor attentions to the patients. The nurses wear a pretty blue and white striped uniform, white aprons, collars and cuffs, and Sister Dora caps. The superintendent nurse is a lady who was trained in one of the large American hospitals, and the pupil nurses are under her immediate control. She holds various classes daily, and gives practical instruction in medical and surgical nursing. Lectures are also given by the doctors upon the staff. Antiseptic surgery is very thoroughly carried out in the Jesu Maria Hospital, even the caps and dresses of the nurses being sterilised before they are permitted to be worn in the operation-room. The nurses are taught to prepare everything that is required for the use of the surgeons, and are very thoroughly drilled in every detail of modern surgical work The attentions of the nurses to the male patients are confined to the giving of medicines, taking of temperatures, and assisting with dressings. A certain number of wards only are under the supervision of the head nurse, and these are characterised by greater order and cleanliness than those which are under lay authority.

### The Diet Question.

The Commissariat Department is entirely in the hands of religious sisters, who are also responsible for the cleaning of the wards, corridors, etc. Great watchfulness has to be exercised by the nurses, as the patients are very apt to exchange food with each other and otherwise evade the doctor's orders. To a Neapolitan it is very appalling to be deprived of his customary macaroni and all the other savoury morsels which even the poorest contrive to secure in their own homes. Necessary ablutions are very often firmly declined by the patients, and unless medical authority intervenes, the nurses are obliged to submit to the inevitable, and forego the satisfaction of washing at least some of their patients.

After Training.

At the end of two years if a nurse passes her examination satisfactorily she receives a diploma, and can then usually obtain constant employment as a private nurse, although she never commands such high fees as her English sisters. Italians would be greatly startled if a nurse demanded two or three guineas a week for her services. As a rule the nurses are not highly educated in Italy; and there is still a great prejudice against nursing as a profession for women of the upper and middle classes. But in the course of time, no doubt that feeling will disappear, and other hospitals will offer facilities to women who desire to earn an honourable living in a country where, at the present moment a single lady may never walk out alone, nor make any attempt to support herself without running serious risk of losing her position in society and scandalising all her male and female relatives.

### The Night Superintendent.

BY AN EX-MATRON.

Perhaps amongst all advertised posts that of night superintendent is the one which most seldom appears in the columns of The Hospital, and when it does it is the one for which there is the least competition. It is evidently not a coveted position, although it presents unique opportunities for observation and work of a specially interesting character, and may be looked upon as a stepping-stone to higher appointments. What is the reason of its apparent unpopularity?

An Isolated Life.

To begin with, night duty is not generally liked. The *bouleversement* nature undergoes in attempting to change night into day; the weary hours spent in courting sleep to the strains of a piano organ, or the pickaxe of the road repairer; the debarring from social pleasures; and last, but not least, the pangs of indigestion to which the night worker so often falls a victim; all these, no doubt, militate against the popularity of a "permanent" post involving a long period of night duty. Next to a matron the night superintendent leads the most isolated of lives. She must be content chiefly with the society of the night nurses; she must, as it were, be with them, and yet not of them. She must be their friend and yet remain their disciplinarian. She must live in close contact with them, but must to a certain extent hold herself aloof. She must treat them all alike, and can have no close personal friend amongst them.

Unacknowledged Services.

Another hardship of her lot is the treatment she is apt to receive from the day sisters. These are a clan in themselves, each as a rule very jealous over her own ward, which she often looks upon as freehold property, deeming the appearance and work of the night superintendent an intrusion. It is the ward sister who is always to the fore with the visiting medical staff, the night sister's face is often unknown to them. The credit for good nursing and good management is likely to be absorbed by the day sister who is always in evidence, while the night sister's share is forgotten, and often unacknowledged by those who should be most recognisant.

Her Silent Reward.

Under the vigilance of the night superintendent come the bad cases in every ward; often numerous, and at a time when the resident doctors are not easy of access, and must not be unnecessarily disturbed. It is she who must decide the significance of a symptom, she who must be ready for any emergency. She must receive the accidents and attend to the casualties; she must always know where she is most needed, and be on the spot whenever she is wanted. With her lies all the responsibility; she must "know," not only "pretend to know." She works for the love of the work itself. She is only too aware how often patients are at their worst when the ward is quiet and dark; that the first night after an operation may be the most critical period; that it is when night is wearing into dawn that vitality is at its lowest, and that these are the opportunities for the truest and best nursing, and thus she reaps her silent reward.

A Woman of Deeds.

She will have ample opportunity too of proving herself loyal to those over her, and in this case will be more likely to receive appreciation. The certainty that all will be well in the wards until morning, that friction will be avoided, and that tact and common sense will be used, makes a matron retire to rest with many a grateful thought for the night superintendent, and she will not grudge her half an hour's cheerful conversation before sending her on duty, or neglect to afford her a meed of praise. But the night superintendent must be one who is above working for praise, and who does not look for gratitude. She is content to realise that while her responsibilities are great, and her privileges comparatively few, her unseen sphere for good is immense. She will be a woman of deeds, not words.

### Appointments.

[No charge is made for announcements under this head, and we are always glad to receive, and publish, appointments. The information to insure accuracy should be sent from the nurses themselves, and we cannot undertake to correct official announcements which may happen to be inaccurate. It is essential that in all cases the school of training should be given.]

Chailey Isolation Hospital, Lewes.—Miss Isa Nicoll has been appointed matron. She was trained at St. Marylebone Infirmary, London, and has since held appointments under the Metropolitan Asylums Board; been night superintendent at Fulham Infirmary, Hammersmith; sister at the London Fever Hospital, and sister and assistant-matron at Delancey Hospital, Cheltenham.

Gorleston Cottage Hospital.—Miss Gertrude E. Blower has been appointed matron. She was trained at Guy's Hospital, London.

Greenock Infirmary.—Miss Maud Sinclair has been appointed sister. She was trained at the Royal Infirmary, Sheffield. She holds the Edinburgh maternity certificate.

Hastings Union Infirmary.—Miss Ethel Sanderson has been appointed assistant nurse. She was trained at the Royal Boscombe and West Hants Hospital by the Meath Workhouse Nursing Association.

St. George's-in-the-East Union Infirmary.—Miss Frances Robinson has been appointed home sister. She was trained at St. George's-in-the-East Infirmary, and has since held the appointments of staff nurse and ward sister.

University College Hospital.—Miss A. Margaret Bird has been appointed night sister. She was trained at St. Bartholomew's Hospital, London.

### Echoes from the Outside World.

### Movements of Royalty.

The King, it is announced, will open the ensuing Session of Parliament in person, and will be accompanied by the Queen. There will be a full State ceremony, and the new procession road down the Mall will be used for the first time.

Tuesday was the birthday of her Majesty the Queen, who was born on December 1st, 1844. As usual the Queen spent the day at Sandringham with the King and members of the Royal family, a small party of personal friends having been invited to share in the domestic festivities in connection with the anniversary. The donors of the large number of presents received by her Majesty included the King and Crown Prince of Denmark, the Tsar and Tsaritsa, the German Emperor, the King of Portugal, and the King of Italy. Between five and six hundred school children on the King's Norfolk estate were entertained by the Queen at tea in honour of her birthday. The King and Queen gave a dinner party in the evening. From all parts of the world, and from all classes of the community, her Majesty received congratulations. Royal salutes in honour of the day were fired in St. James's Park and at the Tower of London; at Edinburgh Castle; at Kingstown in Ireland; and at all saluting stations at home and abroad.

### A College for Devonshire.

The late Mr. Seale-Hayne, M.P., has willed the residue of his large estate to be used for the purpose of establishing a College of Science, Art, and Agriculture for the County of Devon, to be built in the immediate neighbourhood of Newton Abbot, and to be open to students throughout the whole country. Mr. Seale-Hayne had a very fine collection of pictures, and these, if his executors think desirable, are to be hung in the gallery of the institution. It is anticipated that the sum of £150,000 will be handed over for the college.

### Physical Education of Children.

An interesting lecture on "The Physical Education of Children" was delivered on Friday night at West Heath School, Hampstead, by Mr. Eustace Miles, who is well known as a scholar and an athlete. Mr. Miles urged that exercise should be frequent, brisk, and brief, not slow and of a severe nature, entailing a prolonged strain on the system. He strongly deprecated the use by children of spring-grip dumb-bells, and insisted upon the necessity of children learning the art of repose. Children ought also, he contended, to have an opportunity of expressing themselves freely. Above all, he emphasised the supreme importance of exercises in breathing, in trunk-training, in balancing, and in foot movements. Such exercises, carefully and correctly practised, should form the basis of all physical development, otherwise wrong ways of doing most things would be acquired. Children should be taught to take a pride in their bodies—to appreciate the beauty of the body, and to know, not from books, but by observation, the elementary laws regulating its health.

### A Female Advocate in a Murder Case.

At Toulouse last week, for the first time in the history of the French Bar, a prisoner charged with murder was defended by a female advocate. Her client was accused of causing the death of her son-in-law, who was found lying stabbed in five places in the courtyard of a house in Toulouse, and died next day. Mdlle. Dilhan, who was attired in the usual barrister's wig and gown, is only 26 years of age. She is said to have examined and cross-examined the witnesses with great skill; and though she did not succeed in inducing the jury to acquit the prisoner, she secured for her the lowest possible penalty in the case. Instead of a lifelong sentence for culpable homicide being imposed, the court inflicted one of 18 months' imprisonment for aggravated assault.

### A Valuable Pendant Jewel.

Last week there was sold at "Christie's" a pendant jewel of gold set with diamonds, probably of German workmanship, dating from the last years of the sixteenth century. In form it resembles the barge of Cleopatra, manned by two rowers, whilst in the prow and stern are male and female musicians in the costume of the period of the jewel; in the centre of the barge, beneath a canopy, are the figures of Antony and Cleopatra. The jewel itself is decorated with applied gold strapwork, it is enriched with polychrome opaque, and translucent enamels, and it is set with table diamonds and pearls. At a comparatively recent date a brooch pin was added. This jewel was given by Queen Anne, for political services, to Sir George Allardice, M.P. for Kintore and Master of the Mint, an ancestor of the vendor. The bidding for it started at £500, and eventually it was knocked down at £6,500, the purchaser being Mr. C. J. Wertheimer.

### Winter Exhibition of Painters in Water Colours.

There are in the Winter Exhibition of the Royal Society of Painters in Water Colours, which opened on Monday, no fewer than 59 drawings, which were presented by the Society to the King and Queen on the occasion of their Coronation. The drawings, which have been lent by the King for exhibition, are all of the same size, and are, perhaps, on the whole, most noteworthy for elaborate and mechanical finish The most admirable landscapes in the collection include a "Souvenir of Italy," by Sir Ernest Waterlow; a "Wet day," by Mr. George Clausen; a "Berkshire Village," by Mr. R. Thorne-Waite; while Mr. R. Anning Bell's "Surprised," and a drawing by the Princess Louise of a young girl in a charming costume, seated and holding a jug in her hand, are specially excellent. So far as the ordinary attractions of the exhibition are concerned, there is, at any rate, one lady who distinguishes herself. Miss Rose Barton is seen to great advantage in her picture of a red-tiled, gabled cottage in a side street in Chelsea. Miss Clara Montalba also contributes a very good study of scenery entitled "Giudecca." Other pictures which should not be missed are, "The Haunt of the Sea Birds," by Mr C Napier Hemy; "St. Jean du Luz," by Mr. Matthew Hale; "A Sunny Summer Day in the Highlands," by Mr. Robert Allan; and "The Sermon," by Mr. Albert Goodwin.

### Ladies' Hats in Theatres.

At Madrid ladies have rebelled against an edict vetoing the wearing of hats in theatres, which managers in the Spanish capital have attempted to enforce. A duchess set the new regulations at defiance by appearing at the play in the very largest head-gear she could find in the milliner's shops, and refusing to remove it when she was requested to do so. The theatre-hat controversy has also been revived in Paris. There are four theatres in the French capital where neither hats nor bonnets are allowed, and in these—the Opera, the Opera Comique, the Francais, and the Theatre Sarah Bernhardt—the rule is strictly enforced. The director of the Opera Comique says that ladies are now quite reconciled to the rule, and have ceased to object to it. At all the other theatres, however, hats larger than ever are worn. One of the managers in Paris suggests as a means of getting rid of them an official intimation that "no ladies under forty are allowed to wear head-gear," but he has not put his proposal into practice in his own theatre.

### Notes and Queries.

**REGULATIONS.**

**The Editor is always willing to answer in this column, without any fee, all reasonable questions, as soon as possible.**

**But the following rules must be carefully observed :—**

1. **Every communication must be accompanied by the name and address of the writer.**
2. **The question must always bear upon nursing, directly or indirectly.**

**If an answer is required by letter a fee of half-a-crown must be enclosed with the note containing the inquiry.**

*Home.*

(81) Can you kindly tell me of a place where a poor woman can be taken in? She is not helpless, only weak through poverty and privation: her age is about 58, and her relations could pay about 3s. per week towards her maintenance. She could help in light work.—*P. B.*

We are afraid that the workhouse is the best home for the case you mention, unless you can place her amongst people whom you know would be kind to her. You might try an advertisement.

Will you kindly tell me of a Roman Catholic Home where one or two little boys could be placed? The father has deserted them, and the mother has five children to support.—*Sister.*

Apply to the Hon. Secretary, Associated Catholic Charities, 21 Oxford Terrace, Hyde Park, W.

*Jewish Home.*

(82) Can you give me the name of a Jewish home or asylum in or near London, if there is such a place?—*E. M. A. S*

The Home for aged Jews, 23 and 25 Well Street, Hackney, E., might meet the requirements of the case. If unsuitable, apply to the Board of Guardians for the Relief of the Jewish Poor, Middlesex Street, Bishopsgate, E.

*Abroad.*

(83) Will you kindly tell me if there are any nursing institutes abroad which employ nurses on the co-operative system? Do you know of any in Cairo or Madeira?—*J. A.*

There are private nursing institutions in Cairo, but we do not know if they are co-operative. We have no knowledge of any in Madeira.

Would you please let me know the name and address of any London agency or institution which sends nurses abroad? Is there such an institution as "Hollands," and if so, where?—*Delta.*

The Nice Nursing Institute (formerly the Holland Institution) Villa Pilatte, Avenue Désambrois, Nice.

*Brisbane.*

(84) Can you give me any particulars as to private or hospital nursing in Brisbane? I am going there very shortly, and should like to know the best way to obtain work.—*H. K.*

There are five hospitals in Brisbane. Possibly the matron of the General Hospital would give you the necessary information if you applied to her, enclosing stamp for reply.

*Upper Burma.*

(85) Will you kindly tell me if there are any nursing institutions in Upper Burma? Where could I apply for information concerning foreign appointments?—*D. E. M.*

The General Hospital at Mandalay has a nursing staff of a matron and one nurse. Communicate with the Secretary, the Colonial Nursing Association, Imperial Institute, S.W., for information respecting nursing in the Colonies.

*Nursing Card.*

(86) I should be so much obliged if you would kindly tell me what particulars should be printed on a private nursing card, whether "Nurse," or "Miss," is usually put; and what fee I might ask? I have had two years' training and hold the L.O.S. certificate.—*Little Britain.*

You could have on your card "Nurse Britain," just underneath the name, neatly placed, "Trained Nurse with L.O.S. certificate," and your address in the left hand corner. The charge varies according to the class of case. Consult the local doctor.

*Paraffin.*

(87) What would be the best emetic to administer, whilst waiting for the doctor, to a child who had taken paraffin?—*E. C.*

Consult a "Nursery Emergency Chart" which should find a place in every home where there are children.

*Convalescent Home.*

(88) Can you tell me of a convalescent home where a nurse could be received for a few months at small cost? After eight years nursing, the last two being spent in consecutive night duty, she has completely run down and become unnerved. She must do something soon, or she will have to draw out her payments to the Royal National Pension Fund.—*Wearied.*

The nurse should apply to the Benevolent Fund which is attached to the Pension Fund.

I should be very glad to know of an inexpensive nursing or convalescent home where a lady with a weak heart and unable to go upstairs could be received for a few weeks' change.—*R. P. H. S.*

The Erith House Institution for Invalid Ladies of Limited Means, Torquay, seems suitable for this case. There are others, for which consult "Burdett's Hospitals and Charities."

*Maternity.*

(89) 1. Can a nurse with general and monthly training qualify for the L.O.S. certificate without becoming resident in a hospital? 2. Is it yet known if the L.O.S. will be considered sufficient qualification under the new Midwives Act?—*M. T.*

1. Write to the London Obstetrical Society, 20 Hanover Square, W. 2. Certainly.

*Weak Ankles.*

(90) Will you tell me what is the best thing to do for my little boy of 2½ years? His ankles are turning over inside, etc.—*H. E.*

This is a question for a medical man; or take him to the Royal Orthopædic Hospital, 297 Oxford Street, which is free.

Will you kindly say if a nurse with two years' general training in a London hospital could obtain a post as district nurse; if so, would you kindly say how I could best obtain such a post.—*Nurse Hampton.*

Yes. Apply the General Superintendent, Queen Victoria's Jubilee Institute for Nurses, 120 Victoria Street, S.W., or advertise.

*District Nurse.*

(91) Can you tell me the usual salary of a district nurse, and her duties?—*E. W.*

The salary varies in every locality; her duties are to attend the sick poor of her district in their own homes, under the direction of the committee engaging her.

*Boot.*

(92) I should be glad to know the best way to get a boot for a patient suffering from a flat foot (the right one only). She is quite unable to pay anything for it.—*M. A. S.*

Apply to the Surgical Aid Society, Salisbury Square, Fleet Street, E.C.

*Nursing Leprosy.*

(93) Will you kindly tell me address of any place in London where nurses are trained who wish to undertake the care of lepers, either in Robbins Island, or elsewhere.—*Nurse E.*

You might apply to the Secretary, the Mission to Lepers in India and the East, 17 Greenhill Place, Edinburgh.

*Nurses' Co-operation.*

(94) I am anxious to join the Cavendish Street Nurses' Co-operation; would you kindly tell me where to apply?—*Anxious.*

Apply to the Lady Superintendent, the Nurses' Co-operation, 8 Cavendish Street, W.

*Hospital Training.*

(95) I have been probationer in a fever hospital for six months, and I should be glad to know if the Society for Foreign Missions takes probationers.—*E. R.*

The Superintendent of the Mildmay Institutions, Conference Hall, Mildmay Park, N., would probably be able to advise you.

**Standard Nursing Manuals.**

"The Nursing Profession: How and Where to Train." 2s. net; 2s. 4d. post free.

"Nursing: Its Theory and Practice." (Revised Edition). 3s. 6d. post free.

"Elementary Anatomy and Surgery for Nurses." By William McAdam Eccles, M.D.Lond., M.B. 2s. 6d. post free.

"Elementary Physiology for Nurses." By C. F. Marshall, M.D., B.Sc., F.R.C.S. 2s. post free.

"Ophthalmic Nursing." By Sydney Stephenson, M.B., F.R.C.S., 3s. 6d. net; 3s. 10d. post free.

"Nursing in Diseases of the Throat, Nose, and Ear." By P. Macleod Yearsley, F.R.C.S.Eng., M.R.C.S. 2s. 6d. post free.

"Fevers and Infectious Diseases." 1s. post free.

### Everybody's Opinion.

**[Correspondence on all subjects is invited, but we cannot in any way be responsible for the opinions expressed by our correspondents. No communication can be entertained if the name and address of the correspondent are not given as a guarantee of good faith, but not necessarily for publication. All correspondents should write on one side of the paper only.]**

### NURSES AS STEWARDESSES.

"M. W." writes: I have read "Nautical's" letter in this week's *Nursing Section* of The Hospital with great interest, especially as "W.'s" letter two or three weeks ago quite discouraged me. I am most earnestly wanting sea voyages and anxious to take up the work of a stewardess, as I am no longer strong enough for the very active nursing I have done many years; but I am very much alone in the world and have no one to help me. I should therefore be most grateful if one who herself has been a stewardess, and has sisters on the P. & O., could give any further particulars of her experience.

### THE USE OF COLLODION.

"Nurse D." writes: I notice in the current number of The Hospital *Nursing Section* a nurse's remarks, taking exception to collodion being called a "pernicious thing" in the prevention of bed-sores. As it was my paper that called forth the remark, might I draw attention to a sentence in Dr. Watson's "Handbook for Nurses," page 111? It runs:—"When the skin threatens to give way a bed-sore may be averted by painting with flexile collodion and removing all pressure from the area involved." This book is advertised in The Hospital under the heading "Important Nursing Text-books."

[Collodion and flexile collodion are two different things. The remarks made by our Examiner apply to collodion, which when used under the circumstance suggested, is, for the reasons our Examiner gave, a "pernicious thing."—Ed. Hospital.]

### POOR-LAW VACANCIES AND GENERAL HOSPITAL-TRAINED NURSES.

"C. A. E." writes: Will you kindly allow me to say a few words on the subject of "Margaret's" letter? To me it is surprising that Poor-law nurses have not taken this matter in hand long ago. For Poor-law Guardians to select for a responsible post a nurse with a general hospital certificate in preference to an equally qualified nurse trained in one of our large workhouse infirmaries appears to me to be extremely unjust, and is obviously a short-sighted policy, which augurs ill for the nursing of the sick poor. When some years ago the Local Government Board insisted upon the appointment of a three years' certificated superintendent nurse for every infirmary containing over a specified number of beds, and recognised the larger workhouse infirmaries as training schools with power to grant certificates, many intelligent women with a taste for nursing were attracted to this branch of the profession. These, after three years' arduous work and study, find to their chagrin that a certificate from a general hospital is preferred for all the important posts. Now we infirmary nurses hold that a well-trained infirmary nurse is as capable of managing a Poor-law institution as her general hospital-trained sister. The subject is much discussed by Poor-law nurses, who for this reason invariably advise would-be nurses not to train in workhouse infirmaries. On the other hand, can it be that workhouse nursing is becoming popular, and that there is no longer a dearth of workhouse nurses? That, whilst our betters are busily looking for the cause and suggesting remedies, the effect, thanks to the output of the infirmary training schools, is disappearing? If this be the case, are not they themselves innocently contributing towards its reappearance at a later date by tacitly admitting the superiority of the general hospital certificate? The question can scarcely fail to recur in a graver form if the workhouse infirmary nurse is to be outclassed so early in her career.

### For Reading to the Sick.

### "FEAR NOT, FOR I AM WITH THEE."

Draw near, thou reft and drooping Heart, draw near, and lift thy gaze
To Him who yearns with outstretched arms thee from thy grief to raise:

Draw near, and clinging close beneath thy Saviour's bleeding heart,
Tell o'er each throb of that deep woe in which thou hast a part;
Tell o'er each drop of dear life-blood which ebbs for thee so fast,
And all thy weary heart-aching upon that true love cast:
In Jesus' cross and passion is the medicine of thy soul,
Yea, there is Balm in Gilead, and a Healer to make whole.

For where the Cross of Jesus is, is peace, and there alone;
And 'neath that banner of His love He gathereth His own:
And thou who wilt be Jesus', must not grudge thy portion small
In His own bitter chalice, who once for thee drained it all.

*C. S.*

When in loneliness and fear we look around for human sympathy, the yearning of our hearts is understood by One who, in the garden of Gethsemane, entreated of His disciples to tarry with Him while He prayed in agony. And when in the moment of pain and weakness God's loving presence is for a while hidden from us, and in the hour of doubt we seem to stand alone in the dark universe, we are strengthened by One who Himself felt that most awful of desolations as He uttered His dying appeal to His heavenly Father, "My God, my God! why hast thou forsaken me?"

The moment when first, in sadness of heart, we kneel in the solitude of our own chamber, unable, it may be, to express our grief in words, but only feeling the comfort of His presence, is a moment which brings a blessing to our whole lives. When we do not need prayer, when we do not want to ask help, when we only know that He understands. There lies the very germ of love—true, pure, deep, untold, unchangeable love, which will uphold us in death, love which will be our joy in eternity. Distant, indeed infinitely distant, must be human sympathy compared with His, yet is it the same in kind? God grant us each in our measure to strive after and attain it.

Blessed, indeed, shall we be when we can once realise this truth. No loneliness, no dreariness, for He is close beside us; no bitterness, for He understands what we cannot bring ourselves to utter; no weakness, for His loving eye is upon us, and in His glance is strength; no fear, for there can be no morrow of trial apart from Him. It is the lesson of years slowly learnt, often forgotten, but it gives peace long before it is acquired perfectly.—*E. M. Sewell.*

O Strength and Stay upholding all creation,
Who ever dost Thyself unmoved abide,
Yet day by day the light in due gradation
From hour to hour thro' all its changes guide;

Grant to life's day a calm unclouded ending.
An eve untouch'd by shadows of decay,
The brightness of a holy death-bed blending
With dawning glories of the eternal day.

*J. Ellerton.*

## Figures and Tables

**Figure f1:**